# Self-care strategies for medical students: an uncontrolled mixed-methods evaluation of a mind-body-medicine group course

**DOI:** 10.1186/s12909-023-04745-9

**Published:** 2023-10-31

**Authors:** Raphael Scullion, Katja Icke, Tatjana Tissen-Diabaté, Daniela Adam, Miriam Ortiz, Claudia M. Witt, Benno Brinkhaus, Barbara Stöckigt

**Affiliations:** 1 Institute of Social Medicine, Epidemiology and Health Economics, Charité Universitätsmedizin Berlin, corporate member of Freie Universität Berlin, Humboldt-Universität zu Berlin, and Berlin Institute of Health, Berlin, Germany; 2 Department of Pediatric Oncology/Hematology, Charité Universitätsmedizin Berlin, corporate member of Freie Universität Berlin, Humboldt-Universität zu Berlin and Berlin Institute of Health, Berlin, Germany; 3 Institute for Complementary and Integrative Medicine, University of Zurich and University Hospital Zurich, Zurich, Switzerland

**Keywords:** Mind-Body-Medicine, Stress reduction, Mindfulness, Resilience

## Abstract

**Background:**

High stress during medical education and its detrimental effects on student health is well documented. This exploratory evaluation study assesses a 10-week Mind-Body-Medicine student course, created to promote student self-care at Charité Universitätsmedizin Berlin, Germany.

**Methods:**

During 2012–2019, uncontrolled quantitative and qualitative data were gathered from 112 student participants. Outcomes including changes in perceived stress (PSS), mindfulness (FMI/MAAS), self-reflection (GRAS), self-efficacy (GSE), empathy (SPF), and health-related quality of life (SF-12) were measured between the first (T0) and last sessions (T1). Qualitative data were obtained in focus groups at course completion and triangulated with quantitative data.

**Results:**

Quantitative outcomes showed decreases in perceived stress and increased self-efficacy, mindfulness, self-reflection, and empathy. In focus groups, students reported greater abilities to self-regulate stressful experiences, personal growth and new insights into integrative medicine. Triangulation grounded these effects of MBM practice in its social context, creating an interdependent dynamic between experiences of self and others.

**Conclusion:**

After completing an MBM course, students reported reduced perceived stress, increased self-efficacy, mindfulness, empathy and positive engagement with integrative concepts of doctor–patient relationships. Further research with larger randomized confirmatory studies is needed to validate these benefits.

## Introduction

Medical education places high demands on future physicians and healthcare professionals. Its rigour is considered integral to students’ future responsibility for patient care and public health [10, 26]. Starting from pre-clinical years, high workloads and academic stress can put enormous strain on students, compounded by encounters with suffering patients, sickness and death during clinical training [11, 24]. Correspondingly, stress-related mental health issues among medical students are well documented. Associations between academic stress and anxiety were found to be especially pronounced among medical students [16], so were increased rates of burn-out and cynicism [12, 19, 28]. Conversely, empathy as a key trait of medical care was found to decrease over the course of medical education [17, 24].

Meanwhile, a rising interest in student welfare has sparked an increase in mental health resilience interventions [32]. The Mind-Body-Medicine (MBM) course was developed by Dr. Aviat Haramati and Nanci Hadzouk at Georgetown University of Medicine (GUSOM) to facilitate medical students’ self-care ability by promoting self-awareness and resilience. MBM belongs to the wider field of complementary and integrative medicine (CIM). It “focuses on the interactions among the brain, mind, body, and behavior, and the powerful ways in which emotional, mental, social, spiritual, and behavioral factors can directly affect health” [9]. MBM also aims to foster engagement with integrative medicine perspectives on health and healthcare [3].

MBM courses have been evaluated in studies using various self-reported quantitative scores, qualitative surveys, and stress biomarkers. While it was generally found to reduce stress and promote empathy, self-care, and well-being [1, 13, 14, 22, 23, 29, 33], results for respective quantitative measures, such as the Perceived Stress Scale (PSS), were not always consistent across studies [7, 13, 33].

This study aimed to investigate the preliminary effects and possible benefits of a GUSOM MBM group course for medical students at the Charité Universitätsmedizin Berlin (Charité). While previous mixed-method and qualitative evaluation of MBM courses focused on questionnaires [14, 22, 29] or interviews [13], this study combines validated quantitative questionnaires and qualitative focus group interviews to capture impressions of MBM group experiences. Under the premise of an exploratory study, no formal hypotheses were put forward. However, we formulated our assumptions at key points of the study. We assumed that medical students may benefit from MBM course participation by increased mindfulness, as well as empathy, self-efficacy and their ability to reflect on themselves and others. We further assumed that participants may learn to better deal with professional and personal stress, improving their quality of life.

## Methods

### Study design

This study was developed based on uncontrolled internal course evaluations conducted by the Institute of Social Medicine at Charité. We performed an exploratory mixed-method evaluation that combined pre-post within-subject quantitative assessments using questionnaires with qualitative semi-standardized focus group interviews. Focus groups were conducted in the same setting as a typical course session. Study participation was voluntary. The study was approved by the ethics committee at the Charité (EA1/159/12, 05.07.2012, amended 06.11.2019). All data collection, analysis, and storage processes were conducted in compliance with the European Directive of Data Protection guidelines.

### Procedure and participants

The GUSOM model MBM course was first implemented at Charité Berlin in 2010. Ten two-hour weekly sessions introduced students to various MBM techniques, such as mindfulness, guided imagery and physical relaxation techniques (see Table 1 below for a course syllabus). It was offered as an elective at Charité and advertised each semester through a student email list. All medical students were eligible via written application. Exclusion criteria included an ongoing treatment for mental illness and previous course participation.

Each session started with a short meditation and group check-in. All participants, including the two faculty members who acted as course instructors, were invited to share their current state of mind and daily life experiences related, but not limited to, their integration of MBM practice. After introducing and practicing a new MBM technique, the group reflected together on individual experiences with each practice. The session ended with a short closing meditation.

**Table 1:** Course syllabus, by session

1. Introduction, opening meditation, drawing exercise I
2. Autogenic training and bio-feedback
3. Mindful eating and mindfulness meditation
4. Guided Imagery I “Special Place”
5. Guided Imagery II “Inner Guide”
6. Written dialogue exercise
7. Loving kindness meditation
8. Shaking and Dancing
9. Mindful Walking
10. Drawing exercise II

### Quantitative measures and statistical analysis

Quantitative data were collected through printed and online questionnaires. Participants were asked to complete them before the first and after the last course session. Due to the exploratory nature of this study, no primary or secondary outcomes were predefined; furthermore, no sample size calculation was performed.

The 10-item Perceived Stress Scale (PSS) was used to measure the degree to which participants experienced their lives as unpredictable, uncontrollable, and overloaded in the previous month [8, 21]. Self-efficacy, defined as “a sense of personal agency […] to cope with […] difficult demands in life” [31] was measured using the 10-item General Self-Efficacy Scale (GSE). Health-related quality of life (QoL) was measured using the 12-item Short-Form Health Survey (SF−12), comprising two subscales for physical (PCS) and mental (MCS) QoL [35]. The Freiburg Mindfulness Inventory (FMI), a 14-item tool, was used to assess participants’ level of mindfulness, derived from mindfulness meditation and defined as present-oriented/non-identifying attention, accepting/non-judgmental attitude, holistic engagement, as well as procedural/insightful understanding [34]. As some participants may not have had engaged with mindfulness meditation prior to the course, a more neutral, cognition-oriented concept of mindfulness was measured using the 15-item Mindful Attention Awareness Scale (MAAS), which appraises an “open and receptive attention to and awareness of current experience” [4]. The Groningen Reflection Ability Scale (GRAS) measured students’ ability to self-reflect. It captures three factors—self-reflection, empathetic reflection, and reflective communication—in one score and was specifically designed to evaluate medical education [2]. Empathy was measured using the Saarbrücker Persönlichkeits-Fragebogen (SPF), a 20-item, revised German translation of the Interpersonal Reactivity Index (IRI). It conceptualizes empathy as a multifactorial trait with both affective and cognitive components on four subscales: empathic concern (EC), fantasy (FS), perspective-taking (PT), personal distress (PD), and an overall empathy score (ES) [25]. For all instruments implemented, higher scores indicated a higher expression of the examined measure.

Sociodemographic variables and questionnaire scores were analyzed descriptively. Student’s paired t-test was used to investigate changes in the scores of the above-mentioned questionnaires. Due to its exploratory nature, this study does not determine statistical significance; instead, it attempts to draw conclusions based on a wider picture of quantitative and qualitative data, alongside a triangulation of these datasets. Computed two-sided *p*-values, where provided, should be regarded as exploratory and are not meant to imply levels of significance. All quantitative data were analyzed using SPSS (version 1.0.0.1406).

### Qualitative data and analysis

Qualitative data were gathered from semi-structured interviews of focus groups, led by institute members unaffiliated with course facilitation after completion of each full course (see Table 2). The interviews were digitally recorded, transcribed, pseudonymized, and analyzed using the software MAXQDA^®^. The evaluation process implemented methodological aspects of both qualitative content analysis and grounded theory [6, 18], applying a combination of deductive and inductive coding strategies. A framework of main themes was established referring to the interview guidelines and existing scientific literature [13, 14, 29] and deductively applied to the material. Memos were created for each subtheme to map and differentiate their discrete meanings and relations. To improve the quality and validity of the analysis and increase intersubjectivity, all data were critically discussed and redacted by the research team during regular meetings. The evaluation team consisted of one medical student, two data managers, and two physicians trained in qualitative research and as MBM course facilitators. Furthermore, the analysis was discussed, analyzed, and optimized by an interdisciplinary qualitative working group (Qualitative Research Network at the Charité) during multiple sessions.

**Table 2:** Interview guideline for qualitative focus groups

Theme	Questions
Introduction	How was your course experience? What benefits did you derive from it?
Motivation	What was your motivation for course enrolment? Did it change during the course experience?
Course structure	What elements of the course did you especially like/dislike?
Critical feedback	Do you have critical feedback on the course?
Academic value	Did the course affect your medical studies?
Future relevance	Was the course experience meaningful for your future on a personal/professional level?
Closing	Open space for additional comments/feedback

## Results

### Sample and baseline characteristics

This study included quantitative and qualitative data, each gathered from 11 MBM courses conducted between October 2012 and February 2019. However, between 2013 and 2014, quantitative and qualitative data collection was not upheld for two consecutive courses. Demographic characteristics and SF−12 scores were introduced from October 2015 onwards. A total of 112 medical students were included in the quantitative data analysis. Since the first evaluation of demographic characteristics in 2015, there were 48 female (70.1%) and 20 male (29.9%) participants with a mean age of 26.2 years (range = 19–42, SD = 4.9). Qualitative data were collected from 11 focus groups comprising 87 participants (62 females, 25 males), with an average interview duration of 52.8 min.

At baseline, participants scored about one standard deviation above the mean of the standard PSS for German students [21], indicating a high stress load (see Table 3).

Correspondingly, the mean baseline values for the mental subscale of SF−12 were registered below the 25th percentile of the published norms for their age group [36], indicating a reduced psychological QoL.

**Table 3:** Baseline characteristics

Variables	n	Study participants Mean (SD) (n (%))
Age, years	68	26.2 (4.9)
Gender, female (n %)	68	48 (70.1)
GSE	90	2.9 (0.4)
MAAS	90	3.6 (0.7)
FMI	88	35.3 (5.6)
GRAS	89	89.1 (11.8)
PSS	88	19.4 (7.4)
SF-12 PCS	46	52.4 (7.5)
SF-12 MCS	46	42.0 (10.6)
SPF PT	86	14.9 (2.4)
SPF PD	86	11.3 (3.8)
SPF FN	88	14.5 (3.0)
SPF EC	86	16.0 (2.1)
SPF ES	84	45.3 (4.8)

Sample size differences due to later introduction of SF-12, sociodemographic data. Minor sample size differences due to incomplete questionnaires

SD, standard deviation; Δ-mean, mean difference T1–T0

CI, 95% confidence interval upper/lower value

T0, time of first evaluation before the commencement of MBM course

T1, time of second evaluation at the completion of MBM course

GSE, General Self-efficacy Scale lower/upper score limit: 1/4;

MAAS, Mindful Attention Scale; lower/upper score limit: 1/6

FMI, Freiburg Mindfulness Inventory, lower/upper score limit: 14/56

GRAS, Groeningen Reflection Ability Scale, lower/upper score limit: 23/115

PSS, Perceived Stress Scale, lower/upper score limit: 0/40

SF-12 PCS, Short Form Health Survey 12 (physical quality of life subscale); SF-12 MCS, Short Form Health Survey 12 (mental quality of life subscale); lower/upper score limit: 0/100

SPF, Saarbrücker Persönlichkeitsfragebogen—German version of “Interpersonal Reactivity Index” (IRI) with four subscales: SPF PT, SPF Perspective Taking; SPF PD, SPF Personal Distress subscale; SPF FN-SPF, Fantasy subscale; SPF EC, SPF Empathic Concern subscale, all subscale lower/upper score limits 4/20; SPF ES, SPF Empathy score lower/upper score limit: 12/60

### Quantitative findings

An overview of results is provided in Table 4. In the pre-post analysis, participants’ level of perceived stress measured by PSS was found to decrease (PSS: T1 15.3, Δ_T0-T1_−4.1, [CI:−5.3,−2.8]), along with increased self-efficacy (GSE: T1 3.1, Δ_T0-T1_ 0.2, [CI: 0.1, 0.3]). However, there were no meaningful changes in health-related QoL, both PCS or MCS (PCS: T1 54.1, Δ_T0-T1_ 1.8, [CI:−0.8, 4.3] / MCS: T1 43.8, Δ _T0-T1_ 1.8, [CI:−1.3, 4.9]). Participants’ level of mindfulness showed improvements for both measures of mindfulness appraisal (FMI: T1 40.3, Δ_T0-T1_ 5.1, [CI: 4.0, 6.2] / MAAS: T1 4.0, Δ_T0-T1_ 0.4, [CI: 0.3, 0.6]), and an increased reflection ability (GRAS: T1 93.5, Δ_T0-T1_ 4.3, [CI: 1.7, 7.0]). After course completion, participants’ empathy measures showed an increased ability to consider others’ perspectives (PT: T1 15.6, Δ_T0-T1_ 0.6, [CI: 0.1, 1.1]) and experienced lower distress when confronting other people’s suffering (PD: T1 10.5, Δ_T0-T1_−0.8, [CI:−1.5,−0.1]). However, there were no changes in the remaining SPF empathy subscales (FN: T1 14.5, Δ_T0-T1_ 0.01, [CI:−0.4, 0.5] / EC: T1 16.1, Δ_T0-T1_ 0.2, [CI:−0.2, 0.5] / ES: T1 46.2, Δ_T0-T1_ 0.9 [CI:−0.02, 1.8]).

**Table 4:** Quantitative outcomes of the MBM course evaluation (mean and SD)

Score	Time	Mean ( SD)	Δ-mean (95% CI)	*P*-value
GSE (*n* = 90)	T0	2.9 (0.4)		
	T1	3.1 (0.4)	0.2 (0.1, 0.3)	< 0.001
MAAS (*n* = 90)	T0	3.6 (0.7)		
	T1	4.0 (0.7)	0.4 (0.3, 0.6)	< 0.001
FMI (*n* = 88)	T0	35.3 (5.6)		
	T1	40.3 (6.1)	5.1 (4.0, 6.2)	< 0.001
GRAS (*n* = 89)	T0	89.1 (11.8)		
	T1	93.5 (8.7)	4.3 (1.7, 7.0)	0.001
PSS (*n* = 88)	T0	19.4 (7.4)		
	T1	15.3 (6.0)	-4.1 (-5.3, -2.8)	< 0.001
SF-12 PCS (*n* = 46)	T0	52.4 (7.4)		
	T1	54.1 (6.5)	1.8 (-0.8, 4.3)	0.170
SF-12 MCS (*n* = 46)	T0	42.0 (10.6)		
	T1	43.8 (9.8)	1.8 (-1.3, 4.9)	0.247
SPF PT (*n* = 86)	T0	14.9 (2.4)		
	T1	15.6 (2.5)	0.6 (0.1, 1.1)	0.013
SPF PD (*n* = 86)	T0	11.3 (3.8)		
	T1	10.5 (3.4)	-0.8 (-1.5, -0.1)	0.026
SPF FN (*n* = 88)	T0	14.5 (3.0)		
	T1	14.5 (3.2)	0.01 (-0.4, 0.5)	0.963
SPF EC (*n* = 86)	T0	16.0 (2.1)		
	T1	16.1 (2.2)	0.2 (-0.2, 0.5)	0.417
SPF ES (*n* = 84)	T0	45.3 (4.8)		
	T1	46.2 (5.7)	0.9 (-0.02, 1.8)	0.055

Sample size differences due to later introduction of SF-12. Minor sample size differences due to incomplete questionnaires. Abbreviations see Table 3

### Qualitative findings

Our qualitative analysis yielded four distinct main themes: “connections and relationships,” “well-being and stress reduction,” “self-awareness and personal growth,” and “mind-body-medicine in medical education.”

#### Connections and relationships

Students described how social interactions and group dynamics in the course were different from their usual social experiences at university, where the academic rigor and competitive culture of medical education could render them isolated and lonely. They appreciated how the MBM course fostered a non-judgmental, open, and non-discursive communication style, that could hold space for the suffering of others. A further analysis of the group discourse yielded a common pattern of inspiring empathy: Following the example of faculty members during check-ins, students expressed themselves openly and authentically to the group. They described how such acts of self-exposure lead to a new recognition of self in the other, supported by perceived implicit and explicit expressions of authentic interest in the well-being of one another.

*Simply to have two professors sitting here, who opened up [to us] and who also experienced stressful days—that helped me sometimes when I went to class and told myself*, ‘*These people are experiencing the same thing on the other side’[even though] no one [at university] wants to admit what it*’*s really like’.* (FGSS18.F2)

Building on these empathic encounters, students reported how connections formed with other course members inspired them to find new and different ways to encounter and connect with others.

*This safe space […] has played such an important part for me and […]I want to […] encounter other people, strangers, the way that we encountered each other here.* (FGWS18.F3)

Students also reflected on the relationship between patients and themselves as future doctors. They expressed a heightened sense of importance in establishing a doctor–patient relationship grounded in empathy, trust, and mutual recognition. Participants recognized their own therapeutic experiences and vulnerability as tools for establishing trust and authenticity with patients to facilitate healing.

*[Relating to the patient from your own experience] creates a completely different impression than working from book [knowledge].*(FGWS16.F1).

#### Well-being & stress reduction

An analysis of course motivation revealed that students’ desire for improved well-being and reduced perception of everyday life stress was a main motivator for participation. Anxiety was commonly reported among participants, brought on by exam periods, feelings of falling behind on academic achievement in a competitive environment, even causing strain on students’ personal lives. Some students complained of experiencing physical symptoms, such as nausea, tinnitus, high blood pressure, and insomnia.

Students reported an increased awareness of how personal and academic stress affected their overall well-being and the value of practicing self-care. They recounted how practicing MBM exercises allowed them to achieve inner calmness. The gradual introduction of different MBM practices throughout the course was described as a process of “*building their own toolkit*” of techniques for reducing and self-regulating mental distress. Successful implementation of this toolkit produced a sense of empowerment and reduced the feeling of helplessness in the face of stressful situations.

*To make the experience that stress is a state [of mind] that can be changed and not something you have no control over at all*—*that already is a pretty cool thing.* (FGWS14.M2)

Some students remained critical of how the medical field held little regard for MBM and self-care practices and provided limited opportunities for engaging in them. Many students reported difficulties with implementing a sustained self-care practice. Perceived lack of time was the most commonly stated reason, as they struggled to balance their time between overloaded daily schedules and MBM practice.

##### Self-awareness & personal growth

Students commonly described MBM exercises as tools and opportunities for self-reflection and increased self-awareness resulting from a perceived increase in mindfulness of their own emotions and mental states.

*I tried to remember the thoughts that would come up [during meditation] and take them with me. I had the feeling, ‘What’s coming up in my mind there […] is really [what is] concerning me at the moment, even if I do not realize it usually’. And that has helped me a lot.* (FGWS18.PF)

For some students, this process had a real-life impact on how they related to themselves. For example, they made changes to their nutritional and other daily habits or developed a more generally increased sense of self-acceptance. Higher degrees of self-awareness also affected relationships formed by students in their social environment. These changes in relationships with others were mostly based on increased emotional openness, empathic recognition of the other, authenticity, and vulnerability. However, the course also lead to challenging experiences, as some students reported confronting individual emotional struggles or personal problems during MBM practice.

#### Mind-body-medicine in medical education

Learning about MBM and CIM as disciplines of modern medicine was reported as a primary motivation for course enrollment. Some participants sought to acquire proficiency in MBM techniques as tools for their future patient care. Students reported increased knowledge of MBM techniques as a main benefit of the course, emphasizing the value of practical experience. New perspectives on the value of MBM and CIM gained through the course led to what students described as a broader, “more holistic” view of the scope of medical practice and the relationship between healthcare professionals and their patients.

### Triangulation—experiences of self and the other

**Fig. 1:**
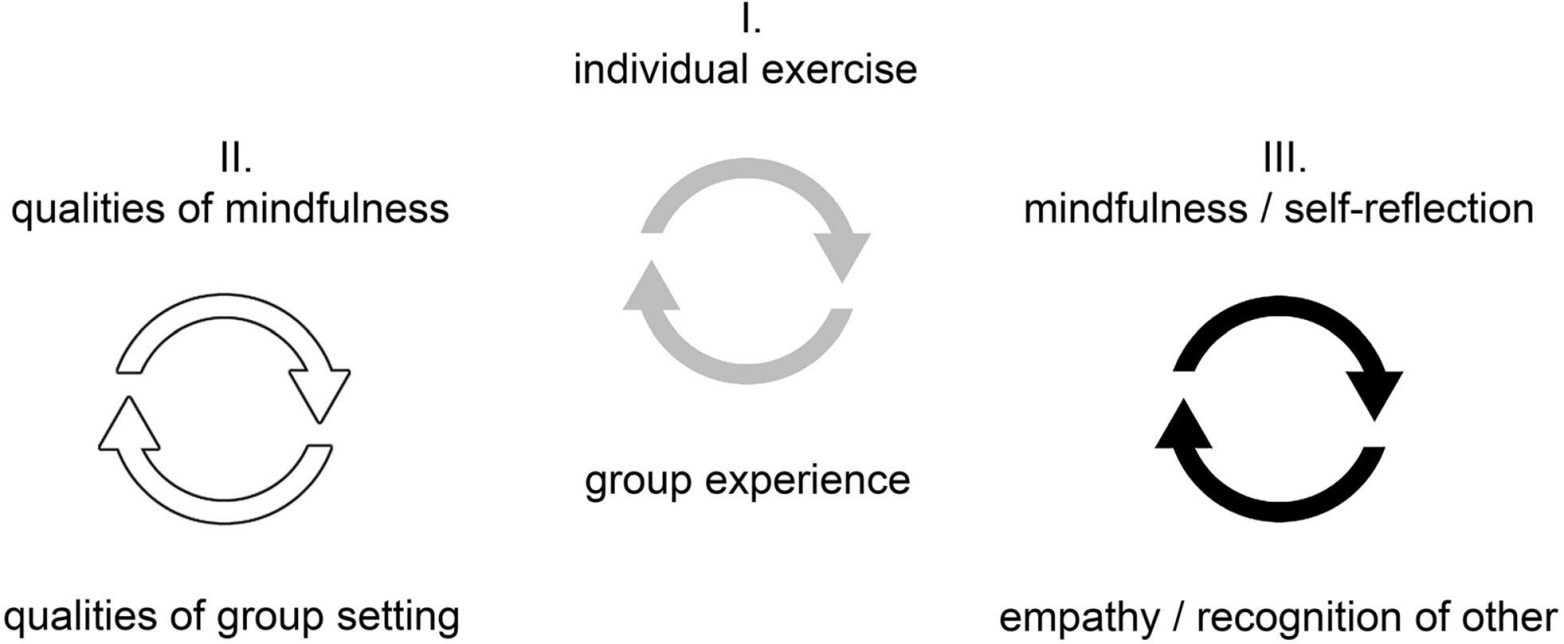
Model for the reciprocative relation between individual and group

Qualitative findings corroborate the quantitative results of increased mindfulness, self-reflection, and empathy, providing a narrative that relates these three outcome values. Students’ accounts of their course experience link their exposure to mindfulness and other MBM practices to increased self-reflection, empathy, and recognition of self in the other. An interdependence between individual and group experiences constitutes the core of these findings, present on three levels. (see Fig. 1):

I. At an organizational level, the MBM course structure alternates between individual exercises and shared group reflections.
II. At a relational level, students` descriptions of group discourse reflect qualities of individual mental states fostered by MBM mindfulness practices such as openness, non-discursiveness and non-judgemental attitude.
III. At a cognitive level, mindfulness practices promote students’ experiences of self-reflection, which create and are in turn created by experiences of empathy, promoted by voluntary self-exposition during group sessions. Recognition of self leads to recognition of the other and, ultimately, recognition of self in the other.

## Discussion

Participants showed improvement across most quantitative measures, including mindfulness, self-reflection, self-efficacy, and perceived stress. However, there were no changes in participant-reported QoL. Empathy, as an ability to take others’ perspective, showed improvement, as did the sense of distress at experiencing empathy. These outcomes were corroborated by focus groups reporting increases in students’ ability to self-regulate stressful experiences and improve their relationships with themselves and others. Participants also recognized the importance of MBM values in the doctor–patient relationship, patient care, and a more holistic view of medicine.

The strengths of this evaluation include its rich dataset, compiled over eight years, and its mixed-methods approach, which allowed for multiple angles of triangulation between quantitative and qualitative data. However, several limitations emerged as a result of its exploratory nature. Under a pre-post evaluation study design, no control group was established. Furthermore, students voluntarily applied to the course, resulting in self-selection bias, and were selected for admission by faculty members. Within these constraints, sample randomization was not possible and participating students may have held certain beliefs or exhibited traits that predisposed them to benefit from MBM programs. Moreover, a continuous collection of data was not upheld for all courses, as quantitative and qualitative data were unavailable for two consecutive courses.

Our qualitative findings indicate that the effects of MBM intervention are deeply embedded in the social framework, discourse, and perspective of its practitioners. Consequently, MBM interventions not only affect measurable outcomes, such as mindfulness, perceived stress, and empathy, but may also influence how students relate to themselves and others, the medical field, and their role as doctors. This supports previous qualitative research conducted by Saunders et al. (2017) at GUSOM, who reported meaningful social connections, self-discovery, and an increased valuing of the doctor-patient relationship as central themes of their study. In this study, data triangulation located a central dynamic of these findings in a reciprocative process between self and the other (see Fig. 1) fostered by meaningful encounters between individual participants and the group.

The qualitative and quantitative findings of this study suggest that an MBM course implemented at Charité may have been suitable for reducing stress and fostering self-care practices among medical students. These improvements were described qualitatively as both immediate benefits from practicing self-care and a gradually gained sense of empowerment, by acquiring a toolkit capable of sustainably self-regulating stress levels.

Our results support those of previous studies on MBM programs for medical students at both American and European universities, which used either PSS [14, 20], distress tolerance [22], or salivary cortisol levels [23] to measure stress reduction. While decreased stress and increased self-care were also among the main results of qualitative MBM course evaluations [13, 14, 29], other quantitative studies could not replicate these effects on stress reduction using the PSS [7, 13, 33].

This study’s qualitative results show that stress relief and self-regulation were primary motivations, but other motivations, such as professional interest in MBM as a future tool for patient care, have also been reported. It is possible that students motivated primarily by personal stress relief benefit from course participation in a different way from others. Divergent study outcomes may thus be a result of their respective group composition.

This study hasn’t shown a meaningful change in MBM course participants’ mental or physical health QoL, as assessed by the SF-12, whereas Esch et al. showed an improvement for mental QoL SF-12 levels in MBM group compared to control [13]. Yet it is possible that the SF-12 is not suitable for use in a sample of generally healthy medical students. The SF-36, and its short version SF-12, were originally developed to assess QoL changes in patients with reduced health [5, 35] and when tested within a sample of healthy patients, the original SF-36 sub-scales, MCS and PCS, were not always found to be independent [27]. Potential MCS changes in our healthy population sample may therefore have been masked.

In her paper on problems with psychometric evaluation of health based QoL, Güthlin [15] expands on the confounding effect of “response shift”. Outcomes of QoL measurements may reflect real changes or they may be the consequence of “response shift” - a cognitive change in the reference system of the patient or changes in the values and concepts held about health and disease [15, 30]. After course participation students reported an increased awareness of the connection between stress, well-being, and self-care practices. However, they also described their difficulties to implement and sustain MBM practices both personally and in the face of a wider academic and medical system often perceived as largely uncaring about self-care practices. Thus, while both qualitative data and a reduction in quantitative stress measure (PSS) support a beneficial effect of course participation on students’ well-being, the overall experience may also have resulted in a shift of values and views that adversely affected QoL appraisal.

In summary, the results of this evaluation showed that after completing an MBM student course, participating medical students experienced reduced perceived stress, increased self-awareness, mindfulness and empathy, as well as positive engagement with integrative concepts of doctor–patient relationships. Further, triangulation of quantitative and qualitative data suggested that these results may be grounded in a dynamic created between individual and group experiences of the course. This dynamic seems to positively affect participants’ relationships towards themselves and others. The results of this study thus suggest that obtaining a deeper understanding of the effects of MBM interventions requires their evaluation in the social context in which they occur. As some dimensions of the group context may remain opaque to purely quantitative studies, a mixed-method approach should be considered particularly suitable for future MBM research. Future studies could conduct more in-depth exploration of these results and validate them in a more rigorous setting, including using control groups such as RCTs.

## Data Availability

Datasets generated and analyzed in the current study are not publicly available to protect participants’ privacy but can be partially accessed upon reasonable request.
